# Post-vaccination myositis and myocarditis in a previously healthy male

**DOI:** 10.1186/s13223-016-0114-4

**Published:** 2016-02-11

**Authors:** Matthew P. Cheng, Michael G. Kozoriz, Amir A. Ahmadi, John Kelsall, Katryn Paquette, Jake M. Onrot

**Affiliations:** Division of Infectious Diseases and Department of Medical Microbiology, Glen site, McGill University Health Centre, 1001 Boulevard Décarie, Room E05. 1811.2, Montreal, QC H4A 3J1 Canada; Department of Radiology, University of British Columbia, Vancouver, BC Canada; Department of Cardiology, University of British Columbia, Vancouver, BC Canada; Division of Rheumatology, University of British Columbia, Vancouver, BC Canada; Division of Internal Medicine, University of British Columbia, Vancouver, BC Canada; Department of Pediatrics, University of British Columbia, Vancouver, BC Canada

**Keywords:** Vaccine, Myocarditis, Rhabdomyolysis, Autoimmune, ASIA

## Abstract

**Background:**

The immunological literature has been redefining clinical phenomena as hypotheses emerge regarding causal links between triggers, immunologic manifestations, and their specific inflammatory cascades. Of late, autoimmune manifestations that appear to be caused by an external adjuvant have been grouped into a complex syndrome referred to as autoimmune/inflammatory syndrome induced by adjuvants (ASIA). This syndrome may present with diverse clinical problems, which may include neurocognitive impairment, inflammatory musculoskeletal changes, and constitutional symptoms. There is evidence in the literature linking vaccines to different auto-immune manifestations. Vaccines have not traditionally been reported to trigger ASIA, although reports are emerging linking the human papilloma virus and hepatitis B vaccines to it.

**Case presentation:**

We report the first suspected case of ASIA in a previously healthy patient who received the Fluad seasonal influenza vaccine, which contains the MF59 adjuvant. He presented to hospital with profound weakness and was diagnosed with severe rhabdomyolysis. He also had elevated troponin-I and extensive cardiac investigations enabled the diagnosis of myocarditis. His infectious and rheumatologic work-ups were negative. He responded well to conservative management and did not require immune suppressive therapy.

**Conclusion:**

Given the benefits of the influenza vaccine, and the low incidence of clinically significant complications, we encourage ongoing seasonal influenza immunization. However, ongoing surveillance is required to evaluate the occurrence of rare adverse events, including ASIA.

## Background

Adjuvants are elements that induce an inflammatory response. In the case of vaccines, adjuvants increase the antigen-specific immune response, to ultimately improve vaccine immunogenicity. This process may also have negative repercussions, inducing autoimmunity. Since 2011, autoimmune manifestations that appear to be caused by an external adjuvant have been grouped into a complex entity referred to as autoimmune/inflammatory syndrome induced by adjuvants (ASIA) [1, 2]. Previously referred to as Shoenfeld’s syndrome, the disorder has been redefined; it is characterized by neurocognitive, inflammatory musculoskeletal, and/or constitutional symptoms upon exposure to an external stimulus, improvement following withdrawal of the offending agent, and may involve the development of autoantibodies, specific HLA phenotypes or evolution into a rheumatological disorder [3]. The exact mechanisms linking external adjuvants to the various immune responses described are not known.

There is longstanding literature linking vaccines to different auto-immune manifestations [4]. In recent years, immunizations [5–7], especially the human papilloma virus and hepatitis B virus vaccines [8], and various vaccine adjuvants [9, 10] have been linked to ASIA. However, the seasonal influenza vaccine is an uncommon trigger for autoimmune disease, and is not commonly associated with rhabdomyolysis or myocarditis. One study showed a tendency for autoantibody production in adults following the influenza vaccine, but without clinical disease correlation [11]. We report the first suspected case of a seasonal influenza vaccine induced-ASIA in a previously healthy male who presented to hospital with simultaneous rhabdomyolysis and myocarditis.

## Case presentation

A 65 year old previously healthy male presented to hospital with profound weakness. Five days before admission, he had received the Fluad seasonal influenza vaccine, comprised of one influenza A H1/N1 virus, one influenza A H3/N2 virus, one influenza B virus, and the MF59 adjuvant. Two days later, he developed bilateral crampy leg pain, muscle tenderness, and progressive weakness. He became unable to weight-bear and was brought to the emergency department by his wife. He denied experiencing headaches, paresthesias, arthralgias, skin lesions or constitutional symptoms. An infectious review of systems was unremarkable. He was not taking any medications or herbal preparations prior to admission, nor did he use recreational drugs.

On physical exam his vital signs were: pulse 92/minute, blood pressure 124/84, respiratory rate 20/minute, oral temperature 36.7 °C, and his oxygen saturation was 100 % on room air. His neurological exam was significant for 4/5 strength in bilateral hip flexors, hip extensors, hip abductors and hip adductors. He could not stand from the sitting position without the aid of his arms. The large muscle groups of his arms and legs were tender. Cardiac examination revealed a jugular venous pressure (JVP) two cm above the sternal angle, a normal apical beat, a normal S1 and S2 without any extra heart sounds, murmurs or rubs. His respiratory exam revealed mild crackles in the left lower lobe. The remainder of the examination was unremarkable.

His blood counts were WBC 11 × 9^9^/L, hemoglobin 123 g/L (MCV 83 fL) and platelets 134 × 10^9^/L. Electrolytes included sodium 126 mmol/L, potassium 3.2 mmol/L, chloride 101 mmol/L, bicarbonate 17 mmol/L, phosphate 0.75 mmol/L and magnesium 0.97 mmol/L. Other laboratory parameters included urea 11.6 mM/L, creatinine 157 mM/L, creatine kinase (CK) 7736 U/L (normal < 150 U/L) and troponin-I 9.44 mcg/L (normal < 0.2 mcg/L). A blood ethanol level was negative. His EKG revealed normal sinus rhythm and a right bundle branch block, without ischemic features. A chest radiograph and contrast enhanced computed tomography scan of the chest revealed a hiatus hernia, left lower lobe opacification, without evidence of pulmonary embolism.

**Fig. 1:**
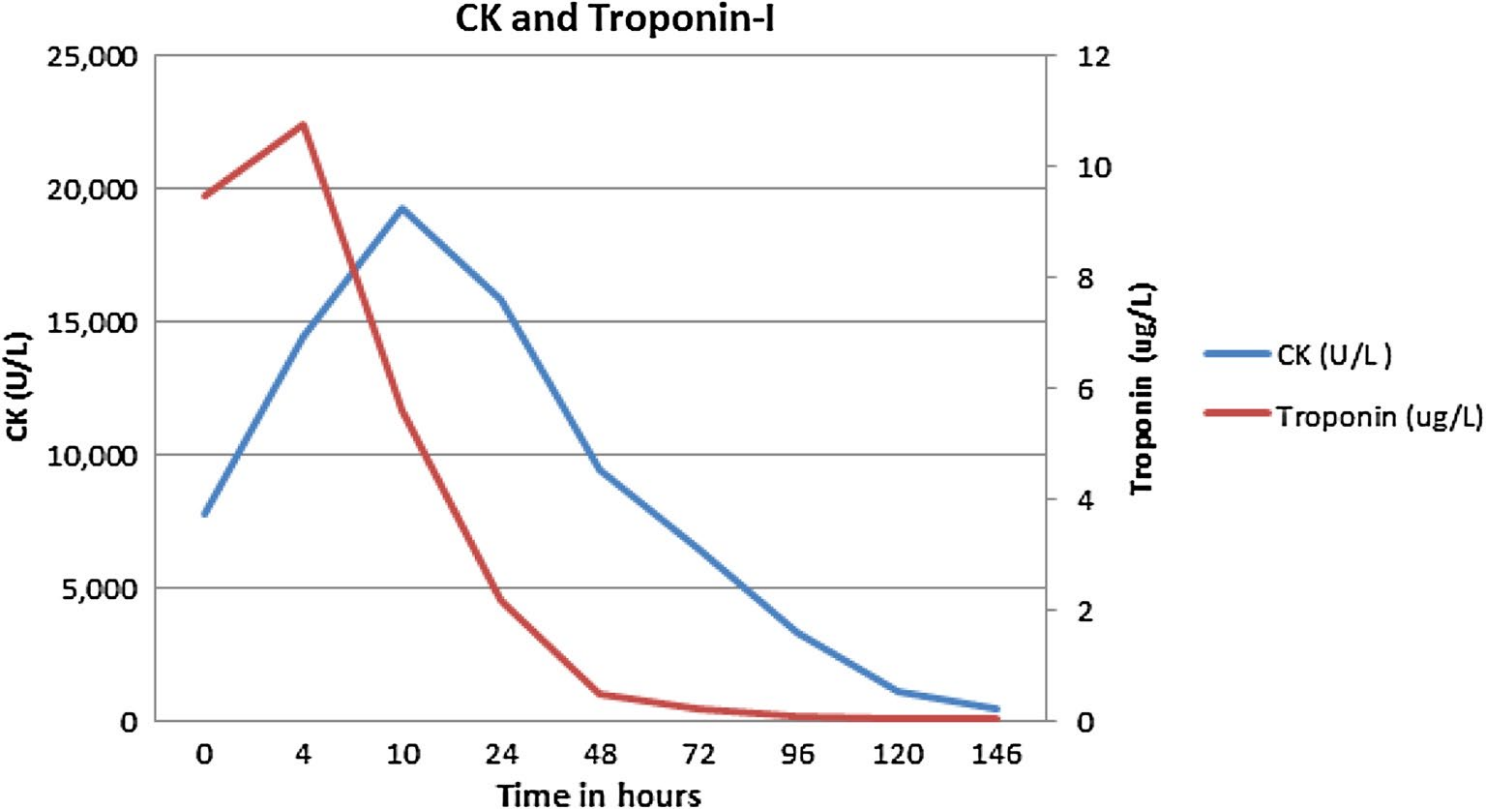
Trend of patient's creatine kinase and troponin-I

Acute rhabdomyolysis was diagnosed and the patient received four liters of fluid over the ensuing 24 h. During volume resuscitation, he developed pulmonary crackles, his JVP increased to 5 cm above the sternal angle, and his oxygen saturation decreased to 90 % on room air. Supplemental oxygen and a single dose of furosemide 40 mg IV were provided, to which he responded well. He also initially received piperacillin–tazobactam for possible left lower lobe pneumonia. No steroids were prescribed. His creatinine, CK and troponin levels trended downwards after 12 h of therapy, and almost normalized within 5 days (see Fig. 1).

A basic immunological workup was within normal limits, with an antinuclear antibody test (ANA) of 1:160, homogeneous pattern (non-specific), and negative anti-dsDNA, ENA panel, C3, C4, rheumatoid factor, and ANCA tests. Serum protein electrophoresis (SPEP) was consistent with an acute phase reaction and urine protein electrophoresis (UPEP) was negative. Dipstick urinalysis revealed 1+ protein and 1+ blood and his urine was positive for myoglobin. Microscopy revealed granular casts, urate crystals and no evidence of red blood cells. HIV, hepatitis B and C serological tests were negative. Blood and urine cultures yielded no growth. Computed tomography scans of the head and lumbar spine were negative; no other cause was found to explain the patient’s weakness. An echocardiogram performed on day three of admission revealed normal biventricular systolic function without regional wall motion abnormalities. Cardiac MRI on day four confirmed the diagnosis of myocarditis. A muscle biopsy was not performed as the patient recovered promptly; he was discharged home on day six.

## Discussion

This patient presented with a gradual onset of weakness and muscular pain. He had received the influenza vaccine 5 days prior to the onset of symptoms. Laboratory investigations indicated rhabdomyolysis, myocarditis, and acute kidney injury, which all resolved by the sixth day of admission.

In rhabdomyolysis, there is breakdown of skeletal muscle cells, resulting in the release of cellular constituents such as electrolytes, myoglobin and cellular enzymes, including creatine kinase. The consequences thereof can include life threatening disseminated intravascular coagulation, electrolyte disturbances, and acute kidney injury. There are numerous known causes of rhabdomyolysis [12, 13]; they are summarized in Table 1.

**Table 1:** Common causes of rhabdomyolysis

Category	Example
Autoimmune diseases	Dermatomyositis and polymyositis
Drugs and toxins	Numerous: including alcohol, cocaine, heroin, fibrates and statins
Electrolyte disorders	Hypokalemia, hypernatremia, hyponatremia, hypophosphatemia, hypocalcemia, hyperosmolarity, ketoacidosis
Endocrine disorders	Hypothyroidism, hyperaldosteronism
Excessive muscle activity	Alcohol withdrawal, exercise, seizures
Genetic disorders	Numerous: including disorders of glycolysis, glycogenolysis, lipid metabolism, mitochondrial pathways and nucleotide metabolism
Hypoxia	Prolonged immobilization, artery occlusion
Idiopathic	
Infections	Viral (coxsackievirus, Epstein–Barr virus, herpes viruses, HIV, influenza A and B) Bacterial (*Clostridium spp., F. tularensis, L. pneumophilia, Salmonella spp., S. pyogenes, S. aureus*) Parasitic (malaria)
Temperature	Heatstroke, malignant hyperthermia, malignant neuroleptic syndrome, hypothermia
Trauma and compression	Crush injury syndrome, electrical injury

Aggressive fluid management is key in avoiding myoglobin-induced oxidative damage to the kidney [13]. This patient was treated with modest fluid resuscitation, due to the concern of circulatory overload in the context of suspected cardiac myositis. He did indeed develop signs of volume overload (increased JVP and respiratory crackles), with favorable response to decreased fluid administration and a diuretic. In addition to the nephrotoxic effects of myoglobin, recent evidence suggests that pro-inflammatory cytokines, chemokines and NLRP3 inflammasomes partake in the pathogenesis of rhabdomyolysis-induced acute kidney injury [14], highlighting the importance for additional research in the management of this condition.

As the history, physical examination, and laboratory markers did not suggest an alternative cause for rhabdomyolysis, we believe that his presentation was most in keeping with ASIA. Furthermore, he fulfilled many of the major suggested criteria for the diagnosis of ASIA (see Table 2). Connective tissue causes of myositis were ruled out by the normal ANA titer, and negative ENA, C3, C4, RA, and ANCA panels. Illness resolution without anti-inflammatory or immunomodulatory medication also goes against connective tissue diseases. The physical examination, SPEP, UPEP, and CT scans were not suggestive of Guillain–Barré syndrome. His cardiac evaluation suggested myocarditis and not myocardial infarction as a cause for the troponinemia. Unfortunately, while the patient’s blood cultures were negative, the patient did not produce any sputum for culture, and a respiratory polymerase chain reaction (PCR) panel to detect common respiratory pathogens was not obtained. Although infectious etiologies of rhabdomyolosis have been previously well described [15–18], we suspect these to be less likely, as the patient denied upper and lower respiratory tract infection symptoms.

**Table 2:** Criteria suggested ASIA diagnosis

	Exposure to an external stimuli (Infection, vaccine, silicone, adjuvant) prior to clinical manifestations
Major criteria	The appearance of ’typical’ clinical manifestations:
	Myalgia, Myositis or muscle weakness
	Arthralgia and/or arthritis
	Chronic fatigue, un-refreshing sleep or sleep disturbances
	Neurological manifestations (especially associated with demyelination)
	Cognitive impairment, memory loss
	Pyrexia, dry mouth
	Removal of inciting agent induces improvement
	Typical biopsy of involved organs
Minor criteria	The appearance of autoantibodies or antibodies directed at the suspected adjuvant
	Other clinical manifestations (i.e. irritable bowel syn.)
	Specific HLA (i.e. HLA DRB1, HLA DQB1)
	Evolvement of an autoimmune disease (i.e. MS, SSc)

For the diagnosis of ASIA, the presence of at least 2 major or 1 major and 2 minor criteria must be apparent. Table reprinted from Journal of Autoimmunity, vol. 36(1), Yehuda Shoenfelda and Nancy Agmon-Levin, ‘ASIA’—Autoimmune/inflammatory syndrome induced by adjuvants, pages 4–8, Copyright 2011, with permission from Elsevier

Few reports propose an association between the influenza vaccine and rhabdomyolysis and/or myocarditis. There are three case reports of rhabdomyolysis following influenza immunization. However, unlike our case, all three patients were taking a statin drug, and none had features of myocarditis. The first two reports describe patients on statin therapy who developed myalgia and progressive weakness within 24 h of receiving the flu vaccine, who were diagnosed with rhabdomyolysis and acute kidney injury [19, 20]. The third published case of rhabdomyolysis was described 1 week post influenza vaccination in a 57 year old renal transplant patient on simvastatin and cyclosporin A [21]. A final case was reported in the literature: a 60 year old gentleman who developed polyarthropathy, orbital myositis and posterior scleritis 10 days after receiving the 1993 Fluvirin vaccine [22]. Given the ocular involvement, he received oral prednisolone and acetazolamide, with dramatic improvement over the next four months.

Shoenfeld et al. [23] suggest grouping different autoimmune manifestations that are seemingly triggered by an external adjuvant into a syndrome complex, referred to as autoimmune/inflammatory syndrome induced by adjuvants (ASIA). Numerous mechanisms have been proposed to explain the interaction between adjuvants, immunogenicity, and autoimmunity. Many researchers postulate that individuals who develop autoimmune phenomena following vaccination have a genetic risk or an underlying disease that activates inappropriate immune responses [1, 7, 24, 25]. ASIA is characterized by a myriad of neurocognitive manifestations, including chronic fatigue, cognitive impairment and amnesia, as well as the development of inflammatory musculoskeletal findings including arthritis and myositis [3]. Our patient’s presentation would be more in keeping with the latter end of the disease spectrum. He fulfills the original diagnostic requirements proposed by Shoenfeld et al. (see Table 2) [23]. Although inflammatory myopathies have been well described following vaccination [25], including macrophagic myofaciitis (MMF) [26], the occurrence of myocarditis has not. Furthermore, our patient did not manifest a locally stereotyped or immunologically active lesion at the site of inoculation, which would argue against MMF. Despite the atypical nature of our patient’s presentation, including cardiac involvement, we believe that his profound inflammatory response may be attributed to the recent vaccine in the absence of other causes, especially given the timing of adjuvant exposure.

As described in a recent systematic literature review and meta-analysis, while local injection site pain and headache are known reactions, serious adverse effects following influenza immunization are rare [27]. Though cases of myositis have been described in adults after hepatitis B or BCG vaccination [28, 29], cases linking the influenza vaccine to myositis without confounding factors are rare. Although an argument can be made regarding the patient’s radiographic opacification as a possible confounder, we are not convinced that the patient had an infectious process. Based on the patient’s clinical and laboratory findings, a presumptive diagnosis of ASIA was made.

As mentioned, there is convincing data in the literature that the influenza vaccine is safe and effective (27). Significant complications are rare. In addition to hand-hygiene and respiratory etiquette, immunization is an effective tool to curb the spread of the influenza virus [30]. Annual vaccination has been shown to protect individuals of all ages from the flu, decreasing rates of emergency room visits, hospitalization, and death [31]. These benefits outweigh the mild adverse effects, prompting many countries to continue wide-reaching annual influenza vaccination campaigns [32].

## Conclusion

We report the case of a 65-year-old previously healthy male who presented to hospital with acute rhabdomyolysis and myocarditis 5 days after the administration of a seasonal influenza vaccine. This may have been due to the adjuvant in the vaccine, although alternate etiologies cannot be eliminated. At this time, the incidence of rhabdomyolysis and myocarditis post vaccination is limited to case reports. ASIA is an emerging clinical entity, with its share of case reports hypothesizing a causal link with vaccines. Given the benefits of the influenza vaccine, especially in the health care setting and in many vulnerable populations, compared to the rarity of this and other putative complications, we encourage ongoing seasonal influenza immunization campaigns. Furthermore, most available seasonal flu vaccines do not contain any adjuvants. Ongoing surveillance to establish the existence of this posited entity and to evaluate its risks should be pursued.

## Consent

Written informed consent was obtained from the patient for publication of this case report.
